# Providing a policy framework for responsible gene drive research: an analysis of the existing governance landscape and priority areas for further research

**DOI:** 10.12688/wellcomeopenres.16023.1

**Published:** 2020-07-20

**Authors:** Delphine Thizy, Isabelle Coche, Jantina de Vries

**Affiliations:** 1 Life Sciences, Imperial College London, London, SW72AZ, UK; 2 Emerging Ag Inc., Calgary, Canada; 3 Department of Medicine, University of Cape Town, Cape Town, 7925, South Africa

**Keywords:** Gene drive, policy, responsible research, synthetic biology, governance, stakeholder engagement

## Abstract

The progress in gene drive research has made the possibility of a future release in the environment probable. This prospect is raising new questions related to the adequacy of the policy frameworks in place to manage and regulate the research and its outcomes responsibly. A number of international mechanisms are exploring how to evaluate this technology. Amongst them, the Convention of Biological Diversity and the Cartagena Protocol, the review mechanisms of the World Health Organisation, and the International Union for Conservation of Nature are offering international fora for dialogue, while regional entities, such as the African Union, are developing specific frameworks to build their preparedness for oversight of gene drive organisms. In this manuscript, we review the existing regulatory landscape around gene drive research and map areas of convergence and divergence, as well as gaps in relation to guidelines for community engagement in gene drive research.

## Introduction

Research on gene drive organisms is not new, but in recent years technical advances, such as in CRISPR, have supported scientific progress to the point where using gene drive organisms for tackling major issues, such as malaria, is now within the realm of possibility ( Feachem *et al*., 2019; Redford *et al*., 2019; World Health Organization, 2019). Gene drive is a naturally occurring phenomenon of biased inheritance, by which a trait can increase in frequency in the progeny, even if there is a fitness effect, through sexual reproduction. In gene drive research, genetically modified organisms contain a transgene that can be passed on up to 100% of their progeny. The prospect of gene drive research moving from a laboratory-only setting to being used "in the field" is now raising questions about the adequacy of the policy frameworks in place to manage and guide the research and its outcomes responsibly ( Evans & Palmer, 2018; James *et al*., 2018; Kuzma *et al*., 2018).

Laboratory-based gene drive research has benefitted from a rich body of peer-reviewed literature and well-established standards for managing biosafety and the integrity of research experiments ( Akbari *et al*., 2015; American Committee of Medical Entomology & American Society of Tropical Medicine and Hygiene, 2003; Benedict *et al*., 2017; Roberts *et al*., 2017). Many of these standards come from previous experience in the contained use of genetically modified organisms, incuding crops, animals bacteria, or viruses. In addition, the institutions where research has primarily been taking place are part of national structures that provide oversight on biosafety, with institutional boards and licensing systems in place.

Partly because of the clarity and familiarity of this environment to researchers, gene drive research has made significant progress in the past four years. The prospect of gene drive organisms being proposed for field evaluation is growing more likely. It is this next phase of research, which involves large ‘cage’ and field-based evaluations, that has raised more questions about governance. Such field-based studies are not without precedent – including genetically modified crops and insects, *Wolbachia*-infected mosquitoes, and biological pest control ( EFSA, 2020)– but nonetheless without an exact equivalent. Whilst existing guidance, for example from the WHO’s *Guidance Framework for testing genetically modified mosquitoes* ( WHO/TDR & FNIH, 2014), the NASEM report “ *Gene Drives on the Horizon”* ( National Academies of Sciences Engineering and Medicine, 2016) and other recent publications ( James *et al*., 2018) offers direction; there are nonetheless many important questions about how field-based gene drive research can be conducted responsibly. In this paper, we aim to identify and illustrate some of these gaps and some of the challenges that planning for field trials of gene drive organisms can pose.

## International governance relevant to gene drive research

There are several international and national frameworks dealing with genetically modified organisms. At the international level, the primary source of guidance is the Convention on Biological Diversity (CBD) and its protocols, which are the main international instruments dealing with genetically modified organisms ( Lai *et al*., 2019). Gene drive organisms are broadly considered to fall under the scope of the Convention and so the provisions of the CBD and its Protocols are applicable, including for cases involving transboundary movement ( CBD, 2017). The most directly relevant component of the CBD is the Cartagena Protocol, which provides an international framework for managing biosafety, by providing legally binding basis to manage the transfer, handling and use of Living Modified Organism (LMOs) – which is the Cartagena Protocol terminology for Genetically Modified Organism – created through the use of biotechnology. Gene drive organisms are considered LMOs and are subject to the provisions of the Protocol ( Australian Academy of Science, 2017; CBD, 2017; Hogervorst *et al*., 2018; Westra *et al*., 2016). As with other international treaties of this kind, the CBD, the Cartagena Protocol and other conventions only apply to the member states that are party to it and that have translated their provisions into national law.

For gene drive organisms which aim to address vector-borne diseases such as malaria, the World Health Organisation also plays an important role through its committees and oversight bodies. These include the Vector Control Advisory Group (VCAG), which provides guidance to developers of new vector control tools “on the generation of epidemiological data and study designs to enable assessment of the public health value of new vector control interventions” ( WHO-VCAG, 2017). It also contributes to WHO’s assessment of new tools and provides advice to two other WHO bodies, the Malaria Policy Advisory Committee and the Strategic and Technical Advisory Group for Neglected Tropical Diseases ( WHO-VCAG 2017). For other products used to fight vector-borne diseases, WHO has issued ‘recommendations’ prior to their use, which enables the products to qualify for funding via the Global Fund, the main mechanism for supporting countries’ purchases of key public health tools. As part of this process of recommendation, WHO has also had a "pre-qualification" process in place to verify that medicines or vaccines could be produced at a certified quality level before they are put on the market ( World Health Organisation (WHO), n.d.). These mechanisms may be applicable in the future to a gene drive technology for public health and are under review to understand how gene drive technologies would be incorporated into the current WHO guidance and review process. WHO is a crucial component of the governance architecture for gene drive technology applicable to health.

Other international bodies and frameworks are also relevant to the governance of gene drive research and use. The International Union for the Conservation of Nature (IUCN) plays an important role from a conservation perspective, albeit offering a form of “softer” power because of its different legal status. IUCN members are States and government agencies, NGOs, Indigenous Peoples' organisations, scientific and academic institutions and business associations. While IUCN does not have the legal weight of a convention or treaty, it is very influential in setting policy directions and best practices ( Bland *et al*., 2019; Stuart *et al*., 2019), and its large membership means that decisions adopted by the members affect a large proportion of the conservation community. Gene drive organisms, as part of a broader conversation on the role of synthetic biology in conservation, have been discussed by IUCN since 2016 ( IUCN World Conservation Congress, 2016). IUCN is poised to adopt a motion on this topic at its next World Conservation Congress in 2021 ( IUCN, n.d.). How the decision is framed in terms of possible risks, benefits, best practices, and other considerations will provide the underpinning framework for how many conservation funders, researchers and practitioners consider gene drive research.

Discussions have also taken place regarding the applications of the provisions of the United Nations Declaration on the Rights of Indigenous Peoples adopted in 2007 ( United Nations, 2007). This discussion emerged as part of the CBD negotiations on synthetic biology and gene drive during the Conference of the Parties (COP) that took place in 2018 in Egypt ( CBD, 2018). A key topic of discussion was whether the concept of Free Prior and Informed Consent (FPIC) or a similar form of consent – from Indigenous People and Local Communities – should be added as a condition before the release of gene drive organisms. In 2018, the final decision (CBD/COP/DEC/14/19) described that such FPIC needed to be sought or obtained “where appropriate” and “where applicable in accordance with national circumstances and legislation”( CBD, 2018). According to the Ad Hoc Technical Expert Group on Synthetic Biology report, the question of Indigenous Peoples’ rights was considered because releases of gene drive organisms could “impact their traditional knowledge, innovation, practices, livelihood and use of land and water” ( CBD, 2017).

Finally, there is a range of instruments and organisations that have more regional relevance. One such instrument is the Aarhus Convention ( United Nations Economic Commission for Europe, 1998), which focuses on public access to information and participation in decision-making about environmental issues. Originally negotiated under the United Nations Economic Commission for Europe, it currently has 47 signatories. While not focused specifically on genetically modified organisms, its provisions regarding access to information and participation could be considered relevant to gene drive research. In particular, the amendment on genetically modified organisms, adopted in 2005 ( United Nations Economic and Social Council, 2005), will increase the relevance of the Aarhus convention for gene drive organisms, if and when it comes into force.

## Governance of gene drive research in Africa

The African region, under the leadership of the African Union (AU), is at the forefront of efforts to guide and regulate gene drive research, for instance through systematically taking stock of existing guidelines and frameworks to check their suitability, and efforts to set up mechanisms to address some challenges such as transboundary movement, which would be the potential consequence of the dispersal of gene drive organisms use for vector control. In particular, in 2017–2018, the AU created a High-Level Expert Panel to examine new technologies of relevance to development in the region. In 2018, the AU identified gene drive for malaria control as a priority area for research for the region, among other technologies ( African Union, 2018a). The report ( African Union, 2018b), and associated AU resolution ( African Union, 2018a), set out priorities for further work in his area and called for African states to increase their participation in gene drive research and ensure readiness for managing such technologies.

This position has led African states to adopt a common position on gene drive research in international negotiations, for example in the CBD, but also spurred efforts to consider the readiness of interested African states to assess and manage possible future field experiments of gene drive organisms. The development agency of the AU, NEPAD-AUDA, has been mandated to provide support to the AU States to build capacity to regulate and oversee gene drive research, including field evaluations ( Glover *et al*., 2018). In line with that mandate, AUDA-NEPAD organised a series of workshops between 2016 and 2018 with national authorities responsible for oversight of GMOs and other relevant stakeholders to build knowledge on gene drive and on the process of problem formulation as the first step in risk assessment ( Teem *et al*., 2019). Furthermore, under the leadership of the Economic Community of West African States, regulators and policy leads have also been working on developing joint guidelines for the review and assessment of gene drive, which may allow key issues, such as transboundary movement, to be taken into account in decision making and help to align regional decision-making on this technology. These initiatives build on other prior efforts to develop shared guidelines and promote joint review of applications for things such as medicines and seeds ( Ndomondo-Sigonda *et al*., 2018).

The call from the AU for African researchers to continue the development of gene drive technologies for malaria control and elimination ( African Union, 2018a) echoes the call from former Minister of Health of Namibia, Richard Kamwi, for Africans to be more central in the discussion and research on gene drive public health interventions ( Kamwi, 2016). Taken together with the stance taken by the AU, it is clear that countries in the region are taking a proactive approach to manage gene drive research and are not waiting for technology transfer but rather anticipating its co-development. Yet at the same time, there are important concerns that African countries are being used (or would be used) to test unproven technologies, in line with “neo-colonial practices” ( African Center for Biodiversity, 2019). This is an important concern which can only be resolved through appropriate regulation and oversight of these new technologies by empowered, critical and knowledgeable national stakeholders including ethics committees, GMO regulators and others; as well as a rigorous stakeholder engagement.

## Challenges in the regulatory frameworks

Taken together, there appear to be considerable resources for governing gene drive research for public health, not only for laboratory research but also for field evaluations. However, there are three important shortcomings in current governance. Arguably the most important relates issues of overlap, inconsistency and coordination between the growing number of organisations involved in and relevant to the governance of gene drive research. As described, these include the CBD, WHO, IUCN, the Aarhus Convention, and possibly others. The organisations involved, and the guidance they develop, have different premises for values and orientations, and this can cause different approaches to the management of gene drive technologies. This risk is most clearly visible between the CBD approach and that taken by the WHO – both of whom currently are the most authoritative and important stakeholders in the governance of gene drive research for public health interventions, but which come to it from different perspectives - the WHO with a focus on public health, and the CBD with a focus on biodiversity conservation.

The CBD and its protocols are framed primarily by the objective of protecting and conserving biodiversity, with the precautionary approach, as defined in the Rio Declaration ^1^, embedded in most of its decisions and documents ( United Nations, 1992). The Cartagena Protocol governs the “transfer, handling and use of living modified organisms resulting from modern biotechnology” and is intended as a protection against potential “adverse effects on the conservation and sustainable use of biological diversity, also taking into account risks to human health”. Under the Protocol, applications for the release of a gene drive organism require the submission of an application to a competent national authority. Applications are focused on demonstrating risk management and do not offer the opportunity to discuss benefits, such as the efficacy of the proposed organism in addressing the targeted health problem. As a result of the focus on risk minimisation and mitigation, including reducing or controlling transboundary movements, the guidance provided by CBD tends to favour field evaluation designs that are more restricted and localised and demonstrating a form of confinement (as one would have done if designing field trials for crops).

On the other hand, WHO and its committees and panels are focused on ensuring the development of effective and safe public health interventions that improve human wellbeing ( WHO-VCAG, 2017) – where the positive health outcome is the prime focus, and risks and benefits to human health and to the environment are assessed jointly. Researchers’ engagement with WHO is focused on discussing what would be considered a robust evidence base for WHO to be able to determine whether a gene drive tool is an effective public health intervention. This generally means seeking field evaluation designs that would need to eventually be on a large scale to be significant and robust enough to demonstrate a positive impact on disease control. The WHO process is also conceived as a guiding process for researchers. The engagement does not involve a ‘one-off’ approval to conduct field evaluations, but rather constitutes an ongoing, iterative process of discussion and feedback over the duration of research and development for a given product.

Whilst these two sources of guidance do not have to be contradictory, they could pose practical challenges about the design of field evaluation of gene drive technologies, as satisfying the dual priorities of minimising risk to the environment while demonstrating large scale positive health impacts requires careful balancing. This technology is not the first case where a coordinated approach has been required. For instance, the implementation of the Nagoya Protocol, whose objective is the fair and equitable sharing of the benefits arising from the utilisation of genetic resources, has challenged how pathogens get shared for medical research purposes without creating risks of inappropriate use ( World Health Organisation, 2019). Similarly, the use of DDT for malaria control has required engagement between the Stockholm Convention on Persistent Organic Pollutants and the WHO ( World Health Organization, 2001). The challenge is that in the absence of an existing dialogue on specific applications of gene drive for health benefits, it is currently left to researchers to be familiar with both sets of processes and requirements and chart a course that reconciles very different philosophies about the risks and benefits of the technologies that they are investigating. Increased awareness and collaboration between the two would help provide researchers with a clearer sense of the requirements and pathways they are expected to follow and how the two interact.

A second important gap in current guidance for field evaluations of gene drive technologies is the absence of guidance relating to appropriate models for consent and community engagement before field evaluations are conducted. The current guidelines and literature are clear about the inadequacy of individual consent for the approval of gene drive organism release because such release does not fit the human subject criteria ( Kolopack & Lavery, 2017; Singh, 2019; WHO/TDR & FNIH, 2014). Because gene drive organisms are “area-wide” in their application, individual residents in an area where a field evaluation is taking place would not be in a position to opt-in or -out as they may do for a drug or vaccine trial. Instead, it is the community as a whole that needs to come to a decision about whether to allow a field evaluation to proceed or not. The current guidelines do not provide a clear framework on how this community acceptance should be sought. On the one hand, entities such as the CBD ( CBD, 2018) only focus on specific groups (Indigenous Peoples) for the consent process. On the other, some countries have made public consultation a mandatory part of their GMO regulatory process ( Burkina Faso, 2006). Furthermore, research groups have been thinking through the issue of community acceptance proactively as well and have started to develop different models for community decision-making ( Kolopack *et al.*, 2015; Neuhaus & Caplan, 2017; Resnik, 2018).

The challenge with this researcher-led development of guidance is that their development is not always inclusive of diverse voices, and there is a risk of bias from the researchers. Such guidelines are also not official guidance that field studies could be audited against. What would be useful is guidance by an authoritative entity such as the AU, national governments or the WHO, outlining principles for community decision-making. This could be done by drawing on official standards for community decision-making used in other sectors, such as for instance in the infrastructure sector ( International Finance Corporation, 2007), or by considering the development of additional guidance if existing standards have gaps ( Hartley *et al*., 2019). Ideally, the guidance would outline clear principles for community decision-making as well as a framework by which citizens and policymakers can assess whether the engagement has been ethical and sufficient for the community and stakeholders to make a decision ( Emerson *et al*., 2017).

Such guidance should consider practical issues such as whose input should be sought– particularly considering the geographical spread of gene drive organisms and their existence across time. It should also offer guidance about the responsibilities for engagement. For instance, it should arguably be the responsibility of the national government to stipulate that community consent must be sought, and possibly to set out criteria that the process should meet. National authorities may set out a requirement for the government to carry out its own public consultation, while also requiring researchers to carry out engagement and obtain consent for the purpose of their research. At a later stage, when considering the technology rollout, the responsibility for rollout is likely to be with governments, and so how consent and support are sought and by whom may then differ. Other actors may also play a role in engagement. Institutional ethics committee already play a role in overseeing the conduct of research, but health workers or teachers should be involved in information dissemination to support informed choices by communities, local authorities could also be sources of information or play a role in monitoring that engagement is done adequately, etc. Establishing clear expectations about processes, roles and responsibilities would help ensure greater transparency for stakeholders about the role of different actors ( Burgess *et al.*, 2018) and define researchers’ responsibilities in engagement and consent. 

Finally, one important gap in the governance landscape around gene drive research relates to questions around benefits for the individuals and communities in which research takes place. Considerable efforts have gone into determining what should be considered benefits in global health research ( Lairumbi *et al*., 2012; Wynberg *et al.*, 2009) – although arguably not so much in relation to public health-based interventions. Gene drive research is likely to take place over periods of years. Though a large part of the research takes place in the laboratory, work in local communities, to gather baseline data and carry out studies, can go on for several years, well before any release of a gene drive organism may occur. As a result, local communities are engaged in the research but may see many years elapse before a field evaluation takes place and even longer before seeing any benefit from the technology being developed. Most current guidance is focused on benefits for short-term projects or in the case of research involving human subjects. It should be clarified what constitutes legitimate and proportionate benefits to communities participating in research programmes, how those can be provided without representing coersive incentives for consent and without creating unsustainable expectations.

## Discussion and conclusion

If gene drive potential usefulness and viability is established with the data from the laboratory and modelling, it will eventually move to field evaluations in Africa. Beforehand, it is imperative to ensure that such research is conducted in a way that is ethical. We outlined the very rich landscape of existing governance that currently guides the design of gene drive experiments. Taken together, these offer important considerations for researchers, but there are also a number of gaps that need to be addressed. Arguably the most important challenge currently relates to reconciling the different approaches taken by the CBD and WHO, which both impact research design.

This is not unique to gene drive organisms, it would be true to other genetically modified organisms used for vector control as they would be equally relevant to both CBD and WHO. For example, the use of sterile male mosquitoes to combat dengue pioneered by Oxitec ( Carvalho *et al*., 2015) would be in the same position. WHO has issued a guidance for the development of genetically modified mosquitoes for disease control ( WHO/TDR & FNIH, 2014), and the modified mosquitoes are subject to the provision of the CBD and must undergo regulatory evaluations and receive regulatory approval at national level before being released outside of a laboratory.

Yet, because gene drive approaches for malaria control aim to develop a tool that is self sustaining, with a modification that would establish itself and persist in the target population of disease vectors, it raises questions ( Mitchell & Bartsch, 2019) about the adequacy of existing frameworks developed with other genetically modified organisms in mind, and heightens once again the challenge of parallel process under CBD and WHO.

It is currently left to researchers to chart a course through this landscape; it would be helpful for these organisations to jointly consider each other’s processes and ensure future decisions take into account each others’ mechanisms and priorities to minimise the risk of contradictory, disjointed or overlapping guidance. The second gap we outlined is the need for guidance on community-based decision-making for the purpose of field evaluations of gene drive technologies. The development of such guidance needs to be inclusive and transparent, representing the voices and views of opponents as well as supporters of these technologies. It needs to set out the principles for community-based decision-making as well as auditable elements that citizens, researchers and policymakers can use to assess whether the engagement has been ethical and sufficient to support informed decision-making. Finally, best practices and guidelines for what can be considered legitimate and proportionate benefits to research communities would help researchers feel confident that their research set up is in keeping with standards for responsible research.

## Data availability

No data are associated with this article.

## Notes

^1^Where there is a threat of significant reduction or loss of biological diversity, lack of full scientific certainty should not be used as a reason for postponing measures to avoid or minimise such a threat.
