# Supplementary material for: Providing a policy framework for responsible gene drive research: an analysis of the existing governance landscape and priority areas for further research
Source: Wellcome Open Res. 2020 Jul 20;5:173. [Version 1] doi: 10.12688/wellcomeopenres.16023.1 (PMC7477640; doi:10.12688/wellcomeopenres.16023.1)
Supplement: Peer review history

# Peer review: Reviewer response for version 1

**DOI:** 10.21956/wellcomeopenres.17577.r40115

**Published:** 2020-09-07

**Authors:** Louise Bezuidenhout

**Affiliations:** 1 Institute for Science, Innovation and Society, University of Oxford, Oxford, UK

This open letter provides a timely review of current e policy frameworks in place to manage and regulate the gene drive research and its outcomes. It highlights the growing range of international and regional organizations involved in populating this landscape, including the CBD, WHO and IUCN. 

 The latter highlights that while the governance landscape could be viewed as providing considerable resources for oversight of both laboratory research and field applications, current shortcomings exist. These relate to overlap, inconsistency and coordination between organizations. I feel that these are important points to raise, and echo challenges in other areas of policy development. 

 I feel that a key take-home message of this paper is the point it raises about researchers currently needing to chart a course through this landscape. Raising such concerns reaffirms that the responsibility of all organizations involved in policy development to ensure that the determination of "right action" does not fall solely within the remit of the individual researcher.

 I think that this letter provides a both a critical oversight of the current status quo, as well as a vision for the future. I feel that it would be complimented by future pieces looking at current structures for whistle-blowing and raising concern. It would also raise the need for further critical studies looking at the infrastructural commitments necessary to ensure that a coordinated governance landscape can be implemented across national boundaries.

Does the article adequately reference differing views and opinions?

Yes

Are all factual statements correct, and are statements and arguments made adequately supported by citations?

Yes

Is the Open Letter written in accessible language?

Yes

Where applicable, are recommendations and next steps explained clearly for others to follow?

Yes

Is the rationale for the Open Letter provided in sufficient detail?

Yes

Reviewer Expertise:

Social science/empirical ethics

I confirm that I have read this submission and believe that I have an appropriate level of expertise to confirm that it is of an acceptable scientific standard.

# Peer review: Reviewer response for version 1

**DOI:** 10.21956/wellcomeopenres.17577.r39930

**Published:** 2020-09-01

**Authors:** Debora C.M. Glandorf

**Affiliations:** 1 Department of Gene Technology and Biological Safety, Centre for Safety of Substances and Products, National Institute of Public Health and the Environment (RIVM), Bilthoven, The Netherlands

This paper gives a good overview of the main challenges encountered in gene drive research for vector control and the work that is going on in this area on international and regional level. The paper also reflects the need for an international approach with respect to the use of this technology, taking into account the potential large scale effects and stresses the need for a responsible approach and community engagement. The paper is well written, and cites the most important publications in a correct way.

 There are some suggestions for improvement of the text.

 Under the heading:

 International governance relevant to gene drive research

 Line 9 ...created through the use of biotechnology. Please include 'modern' before the word 'biotechnology'.

 Line 10...are subject to the provisions of the Protocol. Include 'thus' before the word 'subject'.

 Line 26 'and are under review....'  A suggestion to include 'currently' before review. 

 Line 26- 28. It is stated earlier that WHO makes recommendations on 'products' used to fight vector-borne diseases and not on technologies. Therefore instead of using gene drive technology it seems more appropriate to use the wording 'gene drive products' or 'gene drive mosquitoes'?

 Part on Aarhus Convention

 Line 6...could be considered relevant to gene drive research'' . Please change into something like 'is relevant to gene drive research in case of deliberate release into the environment or placing on the market'. The amendment on GMOs is in force and is therefore also applicable to gene drive organisms, since they are GMOs. The way the sentences are written now, does not seem to be correct.

 Part starting with 'This position as led African states to adopt....'

 Line 10. 'Review and assessment of gene drive'. Clarify if the review and assessment is applicable to the technology or to gene drive organisms (mosquitoes). As I understand, this part of the text is about guidelines for risk assessment of gene drive organisms and not on the assessment of the technology. It is not possible to perform a risk assessment on a technology, an environmental risk assessment can only be done for organisms, in this case gene drive containing organisms. Please clarify this throughout the text.

 Heading Challenges in the regulatory frameworks

 Line 3  ...relates issues of overlap. Include 'to' before 'issues'.

 Text that starts with 'The CBD and its protocols are framed...'

 Line 7. 'Applications are focused on demonstrating risk management...' This is not correct. Applications are for trials with a specific gene drive organism. The national competent authority performs a risk assessment and only in case a risk is identified, can pose risk managements measures. 

 Line 11. Please clarify which guidance of the CBD is meant. Is this the voluntary guidance as developed under the Cartagena protocol? 

 This voluntary guidance, like all other available guidances on environmental risk assessment of GMOs, generally state that in case of uncertainty about  potential adverse effects on the environment, field trials with GMOs are better performed on a small scale and with some sort of confinement. However, if more knowledge is obtained about the interaction of the GMO with the environment, and no adverse effects are observed, trials could be performed on a larger scale and with less (or no) confinement. In this way the trial size can be expanded in a step-wise manner. This step-wise approach may however be challenging for GMOs such as mosquitoes with a gene drive.

 Text starting with '...whilst these two sources of guidance do not have to be contradictory'

 Line 2-3 It is not the dual priority of minimizing risk to the environment on the one hand and demonstrating large scale positive health impacts on the other hand. It is rather

 that field trials that are step-wise increased to collect data on environmental safety have a different design than trials for health impacts, because they are designed to measure completely different aspects.  That should be emphasized here.

 Under 'Discussion and conclusion':

 Line 1:

 If gene drive potential usefulness and viability is established....'.Is here meant 'potential usefulness and potential viablitity of gene drive' or 'gene

 drive potential, usefulness and viability'? This is not clear from the text.

Does the article adequately reference differing views and opinions?

Yes

Are all factual statements correct, and are statements and arguments made adequately supported by citations?

Yes

Is the Open Letter written in accessible language?

Yes

Where applicable, are recommendations and next steps explained clearly for others to follow?

Yes

Is the rationale for the Open Letter provided in sufficient detail?

Yes

Reviewer Expertise:

Areas of expertise: Environmental risk assessment of GMOs, including organisms with engineered gene drives and their policy frameworks. Processes under the CBD and  Cartagena Protocol on Biosafety.

I confirm that I have read this submission and believe that I have an appropriate level of expertise to confirm that it is of an acceptable scientific standard, however I have significant reservations, as outlined above.

# Peer review: Reviewer response for version 1

**DOI:** 10.21956/wellcomeopenres.17577.r40113

**Published:** 2020-08-26

**Authors:** Martin A. Lema

**Affiliations:** 1 National University of Quilmes, Bernal, Argentina

The article is a compact but still very comprehensive review of the state of affairs pertaining to the current debates and policymaking initiatives around the environmental release of organisms containing artificial gene drives.

 It is a very timely article; besides, no other available publications are reviewing this topic. Therefore, I strongly support its indexing.

 The following are suggestions on different aspects that the authors could consider for taking onboard. Still, please note they don´t constitute an objection to any part of the article, and their observance is entirely optional since the authors display in-depth knowledge and sound criteria to portrait the current situation:

- In addition to the CBD-CPB and other fora mentioned in the article, it would be interesting to read about the potential relevance of WTO-SPS(OIE) in establishing regulatory constraints on this technology, *vis-a-vis* precedents such as the WTO-SPS-GMO(Codex/IPPC) panel.
- In regards to the following sentences: “ *As described, these include the CBD, WHO, IUCN, the Aarhus Convention, and possibly others. The organizations involved, and the guidance they develop, have different premises for values and orientations, and this can cause different approaches to the management of gene drive technologies… Arguably the most important challenge currently relates to reconciling the different approaches taken by the CBD and WHO, which both impact research design.*” Since these organizations have different scopes and objectives, the guidance they develop supposedly addresses different aspects. Therefore, it may be useful to illustrate this with a concrete example of guidance clash.
- In regards to the paragraphs beginning with *“This technology is not the first case where a coordinated approach has been required. For instance, the implementation of the Nagoya Protocol,…*” Perhaps the BWC is another example of an international treaty and fora where there is a need for coordinating approaches to control dual-use pathogens and genetic technologies while minimizing disruption to the biotech industry and biomedical research.
- Regarding the section on models for consent and community engagement, it would be valuable to read about past experiences in Brazil and the USA regarding the other example of genetically modified organisms used for vector control that is already mentioned in a different section.
- References to debates regarding transboundary movement issues may perhaps be enriched by explaining if they relate to intentional/unintentional movements and, if possible, to provide some reference on studies describing how realistic the “unintentional” scenario may be.
- As regards to the paragraph containing the phrase “ *Most current guidance is focused on benefits for short-term projects or in the case of research involving human subjects.*”, it would be important to clarify that field trials of gene drives to control human disease vectors are experiments on technologies to suppress a pest population; therefore they don't constitute research on human subjects.
- The article focuses on gene drives for mosquito control, which is probably the more advanced and sophisticated scenario. Nevertheless, the article could be enriched by references to projects for controlling other kinds of pests, and for purposes other than pest control, and a bit of discussion on their particular implications or relevant debates they may rise.

Does the article adequately reference differing views and opinions?

Yes

Are all factual statements correct, and are statements and arguments made adequately supported by citations?

Yes

Is the Open Letter written in accessible language?

Yes

Where applicable, are recommendations and next steps explained clearly for others to follow?

Yes

Is the rationale for the Open Letter provided in sufficient detail?

Yes

Reviewer Expertise:

Regulation of modern biotechnology.

I confirm that I have read this submission and believe that I have an appropriate level of expertise to confirm that it is of an acceptable scientific standard.
